# Adherence to Anti-tuberculosis treatment and treatment outcomes among tuberculosis patients in Alamata District, northeast Ethiopia

**DOI:** 10.1186/s13104-015-1452-x

**Published:** 2015-09-29

**Authors:** Gebrehiwet Tesfahuneygn, Girmay Medhin, Mengistu Legesse

**Affiliations:** Lmlem Karl Hospital, P.O. Box, 07, Maichew, Ethiopia; Aklilu Lemma Institutes of Pathobiology, Addis Ababa University, Addis Ababa, Ethiopia

## Abstract

**Background:**

Non-adherence to tuberculosis (TB) treatment can result in an emergence of new strains, prolonged infectiousness, drug resistance and poor treatment outcomes. Thus, assessment of the level of adherence to anti-TB treatment, treatment outcomes and identifying factors associated with non-adherence and poor treatment outcomes are vital for improving TB treatment adherence and treatment outcomes in the study area. The main objectives of the current study were to assess the level of adherence to anti-TB treatment among patients taking anti-TB drug treatment and to identify factors associated with non-adherence. Whereas, the secondary objectives were to assess treatment outcomes and factors associated with poor treatment outcomes among TB patients previously treated at the health institutions of Alamata District, northeast Ethiopia.

**Methods:**

In a health facility-based cross-sectional study, TB patients who were taking anti-TB drug treatment were interviewed using a structured questionnaire to evaluate level of adherence to anti-TB treatment. TB treatment outcomes were evaluated using data generated from a record review of previous TB patients who were treated at health facilities of Alamata District from January 2007 to June 2012. Adherence data and treatment outcomes data were computerized separately using Epi-Data version 3.1 and analyzed using STATA version 10.0.

**Results:**

Between November 2012 and January 2013, 116 (58.0 %) male TB patients and 84 (42.0 %) female TB patients were interviewed, of whom 77.5 % were new cases, 23.5 % were smear-positive pulmonary TB (SPPTB) cases, 26.5 % were smear-negative PTB (SNPTB) cases and 50.0 % were extra pulmonary (EPTB) cases. The overall adherence rate to anti-TB treatment was 88.5 %. The main reasons for the non-adherent patients were forgetting to take medication, being away from home, drug side effects, being unable to go to the health facilities on the date of appointment and being hospitalized. In the TB treatment outcomes component of the current study, records of 4,275 TB patients were reviewed and the overall treatment success rate was 90.1 %. Two-hundred fifteen (5.0 %) patients had unsuccessful treatment outcomes, of whom 76 (35.3 %) defaulted, 126 (58.6 %) died and 13 (6.1 %) had treatment failure. Significant predictors of unsuccessful treatment outcomes were being positive for human immunodeficiency virus (HIV) infection [adjusted odds ratio (aOR) = 2.1, 95 % CI 1.5–3.0], being SPPTB case (aOR = 3.4, 95 % CI 2.4–4.8), being SNPTB case (aOR = 2.0, 95 % CI 1.5–2.8)], and being re-treatment cases (aOR = 2.6, 95 % CI 1.5–3.7).

**Conclusion:**

In the present study area, there was a high level of adherence to anti-TB treatment and also a high TB treatment success rate. However, still further effort like health education to patient or family is needed to reduce those factors which affect adherence and treatment success rates in order to ensure higher rates of adherence and treatment success than the currently observed in the present study area.

## Background

Tuberculosis (TB) continues to be a major health concern worldwide [1]. In particular, the emergence of drug resistant strains of TB is considered a global threat to the control of TB. Nevertheless, TB is a curable disease if treatment is received quickly and appropriately. Thus, rapid and accurate diagnosis and the use of effective anti-TB treatments are priority tools not only for minimizing morbidity and mortality, but also for mitigating the spread of TB among the population. However, TB patients who are not cured or non- adherent to their treatment not only pose a serious risk both for individuals and community [2], but also present a challenge to effective TB control [3].

Non-adherence to anti-TB treatment may result in the emergence of multidrug resistant TB (MDR-TB), prolonged infectiousness and poor TB treatment outcomes [4, 5]. In previous studies, patients related factors including feeling better, forgetfulness, lack of knowledge on the benefits of completing a treatment course, running out of drugs at home, distance to the health facility, HIV sero-positivity, alcohol abuse, use of herbal medication, stigma and male gender were significantly associated with non-adherence to an anti-TB treatment [6–12]. Non-adherence to an anti-TB drug treatment was also significantly associated with drug side effects, being in the continuation phases of chemotherapy, pill burden, lack of adequate communication with health professionals and lack of family support [9, 10, 12]. A studys done in the southern part of Ethiopia found that re-treatment cases, having a positive smear at the second month of follow-up, smear-negative pulmonary TB, being older than 55 years and being male were significantly associated with unsuccessful TB treatment outcomes [13]. In a study conducted among smear positive TB patients in northern Ethiopia [14],, being older than 40 years of age, having family size of more than 5 persons, being unemployed and being re-treatment cases were significantly associated with unsuccessful treatment outcomes.

Taken together, the above-mentioned studies indicate that different factors have been associated with non-adherence to anti-TB treatment and TB treatment outcomes in different countries as well as in different communities in the same country. Hence, information on the level of adherence to TB treatment, treatment outcomes and identifying specific factors which affect adherence to TB treatment and treatment outcomes in different settings are important to understand specific problems and accordingly design community/population-based appropriate strategies to reduce these problems.

Ethiopia is among the 22 highest TB burden countries (HBCs) in the world [1]. According to hospital statistics data, TB is the leading cause of morbidity, the third cause of hospital admission and the second cause of death in Ethiopia [15]. In Ethiopia, a control strategy for TB was initiated in the early 1960s with the establishment of TB centres and sanatoriums in a few places, which then followed by the direct observation treatment short course (DOTS) program in the early 1990s [15]. Currently, the DOTS health facility coverage is 95 % and the majority (95 %) of the existing health centers/facilities are implementing DOTS-based TB treatment services. In the Tigray Regional State of Ethiopia, DOTS was introduced in 1995 and the program has now been introduced in all hospitals, all health centers and in most of the health posts. Nevertheless, there is little information on patients’ adherence to anti-TB treatment as well as TB treatment outcomes in this Regional State [14]. Therefore, the two components of the current study were (1) to determine the level of anti-TB treatment adherence and to understand factors associated with non- adherence among TB patients who were on treatment during the data collection period, and (2) to retrospectively review records of TB patients and evaluate anti-TB treatment outcomes as well as identify factors associated with poor treatment outcomes in Alamata District of Tigray Regional State of Ethiopia.

## Methods

### Study area and population

The current study was conducted in Alamata District of Tigray Region State, located at a distance of 600 km northeast of Addis Ababa. Based on the 2007 national census conducted by the Central Statistical Agency of Ethiopia (CSA), this District has a total population of 85,403 (42,483 males and 42,920 females) [16]. During the current data collection, the District had one hospital, 6 health centers and 15 health posts, and all of these institutions were providing the DOTS service. Data for the current study was obtained from the attendees or patient records of the hospital and five health centers, namely, Alamata Hospital, Alamata Health Center, Garjale Health Center, Selen Wuha Health Center, Timuga Health Center and Waja Health Center.

### Study design and sample size

A health facility-based cross-sectional study design was used to recruit study participants to assess the level of adherence to anti-TB treatment among TB patients who were receiving their treatment at the health facilities of Alamata District between November 2012 and January 2013. A sample size of 200 was determined to estimate level of adherence to anti-TB treatment assuming 85 % level of adherence [11, 12] with a 5 % margin of error and 95 % confidence. A retrospective cohort study design was employed to assess TB treatment outcomes for the last five and half years (between January 2007 and June 2012).

### Study participants and data collection for the assessment of treatment adherence

All TB patients age over 15 years, who were on anti-TB treatment for at least 1 month during the study period and who were mentally stable, able to communicate, and provide informed consent were eligible for inclusion in the assessment of treatment adherence. A structured questionnaire was used to interview participants to evaluate level of adherence to anti-TB treatment [17]. The questionnaire recorded demographic and socio-economic characteristics of the study participants, general information about TB, information on treatment adherence and problems associated with non-adherence. The questionnaire was prepared in English and translated into the local language (Tigrigna). Trained data collectors at the respective health facilities interviewed the participants in their local language. The questionnaire was pre-tested and cheeked for clarity and consistency. During the data collection, the completed questionnaires were reviewed and checked for completeness, accuracy and consistency. Patients’ knowledge about TB was ascertained based on their responses to 11 questions posed during a face-to-face interview. To generate variables to be used to evaluate the level of overall knowledge of respondents, all responses to the 11 questions were coded (i.e. correct answer was coded as 1 and incorrect answer was coded as 0) and added mathematically. Those respondents who scored equal to and/or above the mean were considered to have a high level of overall knowledge (or to be knowledgeable) of TB, while those scoring below the mean were considered to have low levels of overall knowledge (or be Non- knowledgeable) of TB.

### Data collection for the assessment of TB treatment outcomes

Treatment outcomes were investigated by reviewing the records of all TB patients who were diagnosed with active TB as defined by WHO criteria and who had received anti-TB treatment between January 2007 and June 2012 using a data collection sheet. The data collection sheet contained socio-demographic characteristics of the study participants and information on treatment outcomes (cured, treatment completed, defaulted, died or treatment failed).

### Description of outcome variables

The outcome variables are (1) adherence to anti-TB treatment as reported by patients attending health facilities for their regular anti-TB medication during a face-to-face interview, and (2) TB treatment outcomes as determined through a review of patents’ records for the past 5 years and 6 months. The overall adherence to anti-TB treatment was determined based on the self-report of the study participants (i.e. the study participants were asked to report the total number of days they missed the prescribed medication/anti-TB tablets) for the last 30 days. The adherence percentage was calculated as the number of doses taken by the respondent as prescribed by the clinician, divided by the number of doses prescribed to the respondent in the last 30 days and then multiplied by 100. Those study participants who took at least 95 % of the medication as prescribed by the clinician were considered to be adherent to anti-TB treatment in the last 30 days, while those who took less than 95 % of the medication were considered as non-adherent to anti-TB treatment in the last 30 days. Moreover, non-adherence was evaluated as the doses of medication missed in the previous day, in the past 3 days and in the past 7 days. Treatment outcomes were assessed using a retrospective record review of patients, based on the WHO and national guidelines definition for TB treatment outcomes and monitoring [18]. For the purpose of modeling, treatment outcomes were dichotomized in such a way that cured and treatment completed patients were placed in one category and all other possibilities were clustered in another category.

### Data processing and analysis

Data collected from participants of the anti-TB adherence study and data collected from the retrospective record review of previously treated TB patients were double-entered into two different files using EpiData software, version 3.1 (Odense, Denmark: The Epidata Association, 2003). The two data sets were analyzed independently using STATA version 10.0 (StataCorp, College Station, TX, USA: Stata Corporation 2007). The effects of predefined potential risk factors on the likelihood of adherence were modeled using logistic regression, and odds ratios with corresponding 95 % confidence intervals were reported as the measures of the degree of associations. Variables significantly associated with the likelihood of treatment adherence in the bivariate analysis (i.e. *P* value <0.05) were included in a multivariable logistic regression model to determine their adjusted relative contributions in predicting the likelihood of treatment adherence. Using the retrospectively extracted treatment outcomes and other patient factors, similar procedures were followed to identify variables which were significantly associated with the likelihood of having unsuccessful treatment outcomes while simultaneously adjusting for the effect other variables. Results were reported as being statistically significant whenever p value was less than 5 %.

### Ethical approval

Ethical approval was obtained from the Institutional Review Board (IRB) of Aklilu Lemma Institute of Pathobiology, Addis Ababa University. Permission was obtained from Tigray Regional Health Bureau and Alamta District Health Office before starting the study. Since the majority (68 %) of the study participants were illiterate, verbal consent was obtained before interviewing each participant, and parental consent was obtained for participants under 18 years of age.

## Results

### Background characteristics of participants who participated in the anti-TB treatment adherence study

Background characteristics of the participants who participated in anti-TB treatment adherence study and the associations of these characteristics with the likelihood of adherence to anti-TB treatment are summarized in Table 1. A total of 200 patients (58 % males, age ranged 15–78 years, mean age, 34.8 years) were interviewed. The majority (77.5 %) of the participants were new TB cases, while 47.0 % were in the intensive phase of treatment. About 89.0 % of the patients knew their HIV status, of whom 14.6 % were HIVsero-positive. With respect to collecting anti-TB medication from health center/hospital, 130 (65.0 %) respondents said that they get their medication daily, 17 (8.5 %) mentioned that they collect their tablets weekly and 53 (26.5 %) said that they collect their tablets monthly.

**Table 1:** Association of background characteristics of study participants (n = 200) and level of adherence to anti-TB treatment

Characteristics	Response categories	Number (%) of respondents	Adherence status		Crude OR (95 % CL)	P value
			Number (%) non-adhered	Number (%) adhered (%)		
Sex	Male	116 (58.0)	15 (12.9)	101 (87.1)	1.41 (0.6, 3.5)	0.46
	Female	84 (42.0)	8 (9.5)	76 (90.5)	Reference	
Age (year)	15–24	58 (29.0)	10 (17.2)	48 (82.8)	5.1 (1.3, 24.5)	0.04
	25–34	54 (27.0)	8 (14.8)	46 (85.2)	4.3 (0.9,21.1)	0.08
	35–44	37 (18.5)	3 (8.1)	34 (91.9)	2.2 (0.3, 13.6)	0.41
	45+	51 (25.5)	2 (3.9)	49 (96.1)	Reference	
Marital status	Unmarried	63 (31.5)	11 (17.5)	52 (82.5)	2.3 (0.6, 8.7)	0.24
	Married	102 (51.0)	9 (8.8)	93 (91.2)	1.0 (0.3, 4.1)	0.96
	Other	35 (17.5)	3 (8.6)	32 (91.4)	Reference	
Treatment history	New cases	155 (77.5)	7 (10.3)	148 (89.7)	Reference	
	Re-treatment cases	35 (17.5)	6 (17.2)	29 (82.9)	1.8 (0.7, 5.0)	0.26
Education	Illiterate	121 (60.5)	9 (7.4)	112 (92.6)	Reference	
	Read and write	20 (10.0)	3 (15.0)	17 (85.0)	2.2 (0.5, 8.9)	0.27
	Primary (1–8)	33 (16.5)	7 (21.2)	26 (78.8)	3.4 (1.1, 9.8)	0.03
	Secondary and above	26 (13.0)	4 (15.4)	22 (84.6)	2.3 (0.6, 8.0)	0.21
Distance home-clinic (km)	0–5 km	92 (46.0)	9 (9. 8)	83 (90.2)	Reference	
	>5 km	108 (54.0)	14 (6.5)	94 (93.5)	1.4 (0.6, 3.3)	0.48
Residence area:	Urban	51 (25.5)	8 (15.7)	43 (84.3)	1.7 (0.7, 4.2)	0.28
	Rural	149 (74.5)	15 (10.1)	134 (89.9)	Reference	
Family size	1–5	138 (69.0)	15 (10.9)	123 (89.1)	Reference	
	>5	62 (31.0)	8 (12.9)	54 (87.1)	1.2 (0.5, 3.0)	0.67
Form of TB	PTB	100 (50.0)	14 (14.0)	86 (86.0)	1.7 (0.8,4.0)	0.27
	EPTB	100 (50.0)	9 (9.0)	91 (91.0)	Reference	
Means of transportation	Foot	118 (59.0)	18 (15.2)	100 (84.8)	2.8 (1.0,7.8)	0.05
	Vehicle	82 (41.0)	5 (6.1)	77 (93.9)	Reference	
HIV status	Positive	26 (13.0)	7 (26.9)	19 (73.1)	3.4 (1.2, 9.3)	0.02
	Negative	152 (76.9))	15 (9.9)	137 (90.1)	Reference	
	Unknown	22 (11.0)	1 (4.5)	21 (95.5)	0.4 (0.1, 3.5)	0.43
Alcohol use	Yes	36 (18.0)	8 (22.2)	28 (77.8)	2.8 (1.1, 7.3)	0.03
	No	164 (82.0)	15 (9.2)	149 (90.8)	Reference	
Family income	<501 birr	111 (55.5)	10 (9.0)	101 (91.0)	Reference	
	≥501 birr	89 (44.5)	13 (14.6)	76 (85.4)	1.7 (.8, 4.2)	0.22
Chewing khat	Yes	10 (5.0)	4 (40.0)	6 (60.0)	6.0 (1.6, 23.2)	0.01
	No	190 (95.0)	19 (10.0)	171 (90.0)	Reference	
Smoking	Yes	9 (4.5)	4 (44.4)	5 (55.6)	7.2 (1.8,29.3)	0.01
	No	191 (95.5)	19 (9.9)	172 (90.1)	Reference	
Knowledge of TB	Knowledgeable	73 (36.5)	8 (11.0)	65 (89.0)	Reference	
	Non-knowledgeable	167 (63.5)	15 (11.8)	112 (88.2)	1.1 (0.4, 2.7)	0.86

*PTB* pulmonary TB, *EXPTB* extra pulmonary TB

The average delay time before being served at the health institution was 10.2 min (SD = 11.3) and 187 (93.5 %) respondents reported that they were satisfied by the services of the health institution. Seventy-four patients (37.0 %) said that they were counseled on each visit, 74 patients were never counseled, 98.5 % of the study participants had open communication with the health providers, and 99 % of the study participants said that the care providers provided privacy for them during visiting the health facility.

Forty-eight participants (24.0 %) had no information on the causative agent of TB and 38 participants (19 %) attributed the cause of TB to a bacteria/germ. The remaining respondents suggested dust (20.0 %), stress or work load (16.5 %), chill (13.5 %) and smoking (7 %) as causative agents of TB. The majority (87.5 %) of the study participants knew that TB is a transmittable disease and 50.0 % were able to mention at least two symptoms, with cough being the most identified symptom (54.5 %). When participants were asked about the consequence of interrupting or stopping taking anti-TB drugs, 73(36.5 %) mentioned death. When the study participants were asked about the curability of the disease, 99 % of the respondents reported that TB is a curable disease. But when they were asked about the duration of TB treatment, only 84.5 % reported the correct duration of TB treatment. Based on their response to the questions, 73 (36.5 %) of the respondents were categorized as having a high level of knowledge about TB.

### Level of adherence to anti-TB treatment

**Fig. 1:**
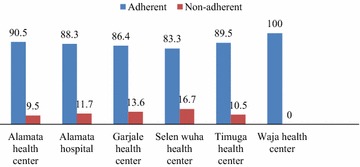
Adherence (expressed in percent) across health facilities in Alamata District. Adherence was measured by self-report and it is based on the ratio of the number of doses taken as per instruction divided by the number of dose prescribed over the last 30 days

The level of adherence to anti-TB treatment was 96 % with the last 3 days, 98.5 % within the last 7 days and 88.5 % within the last 30 days. Non-adherence within the last 30 days was 44.4 % among smokers, 40.0 % among khat chewers, 26.9 % among those HIV sero-positive and 22.2 % among frequent alcohol consumers (Table 1). Relatively, re-treatment cases were less adherent to anti-TB treatment (17.2 %) compared to new cases (10.3 %). The highest proportion of non-adherence within the last 30 days was observed among patients who received their treatment at Selen Wuha Health Center when compared to patients who attended their treatment at the rests of health centers (Fig. 1). The reasons reported for non-adherence were forgetting to take their medication (n = 8; 34.8 %), spending time away from home (n = 6; 26.1 %), failure to go to health facilities on the exact day of appointment (n = 3; 13.0 %), drug side effects (n = 1, 4.4 %), being admitted to a hospital (n = 4; 17.4 %) and other personal reasons (n = 1; 4.4 %).

### Factors associated with non-adherence to anti-TB treatment

Results from logistic regression analysis, which was carried out to assess the associations of selected socio-demographic and other risk factors and the likelihood of non-adherence to anti-TB treatment are summarized in Tables 1 and 2. In a univariable logistic regression analysis, being younger than 24 years of age, HIV sero-positivity, frequent alcohol consumption, having a smoking habit, having a khat chewing habit, a lack of satisfaction with health services and prolonged waiting time at the health facility to get medical services were significantly associated with non-adherence to anti-TB treatment. In a multivariable logistic regression model, being younger than 24 years and prolonged waiting time at health facilities before getting medical attention maintained marginal statistical significance.

**Table 2:** Association of service provider and health facility related factors with adherence to TB treatment (n = 200)

Characteristics	Response categories	Treatment adherence status		Crude OR (95 % CL)	P value
		No. non-adhered (%)	No. adhered (%)		
Patient satisfaction by health service	Yes	18 (9.6)	169 (90.4)	Reference	
	No	5 (38.5)	8 (61.5)	5.9 (1.7, 19.8)	0.004
Health education provided during treatment	Yes	18 (10.3)	157 (89.7)	Reference.	
	No	5 (20.0)	20 (80.0)	2.2 (0.7, 6.5)	0.16
Taking monthly appointment card	Yes	18 (9.8)	165 (90.2)	Reference	
	No	5 (29.4)	12 (70.6)	3.8 (1.2, 12.0)	0.02
Counseling about TB treatment	First visit	8 (9.5)	76 (90.5)	1.2 (0.4, 3.6)	0.76
	On each visit	6 (8.1)	68 (91.9)	Reference	
	Once a while	2 (11.1)	16 (88.9)	1.4 (0.3, 7.7)	0.67
	Never counseled	7 (29.2)	17 (70.8)	4.7 (1.4, 15.7)	0.01
Delay time before served	1–10 min	12 (7.8)	141 (92.2)	Reference	
	11+ min	11 (23.7)	36 (76.6)	3.6 (1.5, 8.8)	0.01
Open communication with health providers	Yes	22 (11.2)	175 (88.8)	Reference	
	No	1 (33.3)	2 (66.7)	0.3 (0.1, 2.9)	0.27
Frequency of dose he/she takes	Daily	19 (14.6)	111 (85.4)	4.4 (0.1, 19.5)	0.05
	Weekly	2 (11.8)	15 (88.2)	3.4 (0.4, 26.2)	0.24
	Monthly	2 (3.8)	51 (96.2)	Reference	
Privacy by health providers	Yes	22 (11.1)	176 (88.9)	8.0 (0.5, 132.5)	0.15
	No	1 (50)	1 (50)	Reference	

### Background characteristics of study participants included in TB treatment outcomes

Between January 2007 and June 2012, a total of 4275 patients (59 % males), with a mean age of 31.7 years, were registered to initiate anti-TB treatment at the study health facilities. The background characteristics are summarized in Table 3. The majority (97.4 %) were new cases, while 589 (13.8 %), 1249 (29.2 %) and 2437 (57.0 %) were registered as SPPTB, SNPTB and EPTB cases, respectively. Among 2864 patients (67 %) who were tested for HIV, 587 (20.5 %) were sero-positive and HIV infection was significantly higher among urban than rural TB patients (17.8 versus 10.2 %, P < 0.001).

**Table 3:** Anti-TB treatment outcomes for five consecutive years stratified by selected background characteristics (n = 4275)

Characteristics	Possible values	Treatment outcomes, n (%)						
		Cured	Treatment completed	Died		Treatment failure	Defaulted	Transfer out
Sex	Male	312 (12.4)	1951 (77.4)	70 (2.7)		8 (0.3)	49 (2.0)	132 (5.2)
	Female	179 (10.2)	1411 (80.5)	56 (3.2)		5 (0.3)	27 (1.5)	75 (4.3)
Age	0–14	20 (3.5)	495 (87.6)	13 (2.3)		0 (0.0)	10 (1.8)	27 (4.8)
	15–24	164 (16.0)	776 (75.8)	15 (1.5)		5 (0.5)	19 (1.8)	45 (4.4)
	25–34	137 (13.5)	764 (75.4)	34 (3.4)		4 (0.4)	18 (1.8)	56 (5.5)
	35–44	83 (12.0)	540 (77.5)	26 (3.7)		3 (0.4)	15 (2.1)	30 (4.3)
	45+	87 (8.9)	787 (80.6)	38 (4.0)		1 (0.1)	14 (1.4)	49 (5.0)
Residence	Urban	251 (12.2)	1628 (79.3)	64 (3.2)		9 (0.4)	29 (1.4)	72 (3.5)
	Rural	240 (10.8)	1734 (78.0)	62 (2.8)		4 (0.2)	47 (2.1)	135 (6.1)
Form of TB	Smear positive PTB	491 (83.4)	0 (0.0)	30 (5.1)		13 (2.2)	16 (2.7)	39 (6.6)
	Smear negative PTB	–	1118 (89.5)	52 (4.2)		–	27 (2.2)	52 (4.2)
	EPTB	–	2244 (92.0)	44 (1.8)		–	33 (1.4)	116 (4.8)
Treatment history	New cases	454 (10.9)	3313 (79.5)	117 (2.8)		7 (0.2)	71 (1.7)	203 (4.9)
	Retreatment cases	37 (33.6)	49 (44.5)	9 (8.3)		6 (5.5)	5 (4.5)	4 (3.6)
HIV status	Positive	90 (15.3)	406 (69.2)	39 (6.6)	3 (0.5)		10 (1.7)	39 (6.7)
	Negative	244 (10.7)	1813 (79.8)	45 (2.0)	6 (0.3)		50 (2.2)	114 (5.0)
	Unknown	157 (11.0)	1143 (80.7)	42 (3.0)	4 (0.3)		16 (1.2)	54 (3.8)
Year of enrollment	2007	81 (12.6)	514 (80.0)	21 (3.3)	2 (0.3)		5 (0.8)	19 (3.0)
	2008	81 (9.6)	673 (80.1)	33 (4.0)	1 (0.1)		10 (1.2)	42 (5.0)
	2009	87 (9.8)	713 (79.9)	20 (2.2)	1 (0.1)		15 (1.7)	56 (6.3)
	2010	107 (13.4)	603 (75.6)	19 (2.4)	1 (0.1)		14 (1.7)	54 (6.8)
	2011	102 (12.9)	614 (77.7)	23 (2.9)	7 (0.9)		17 (2.2)	27 (3.4)
	2012	33 (10.5)	245 (78.3)	10 (3.2)	1 (0.3)		15 (4.8)	9 (2.9)
Health facilities	Alamata hospital	248 (10.1)	1929 (78.8)	60 (2.4)	5 (0.2)		44 (1.8)	164 (6.7)
	Alamata HC	139 (15.4)	725 (80.5)	13 (1.4)	1 (.1)		4 (0.5)	19 (2.1)
	Garjale HC	24 (7.6)	257 (81.9)	13 (4.1)	0 (.0)		13 (4.1)	7 (2.3)
	Timuga HC	59 (12.4)	351 (73.7)	31 (6.5)	7 (1.5)		13 (2.7)	15 (3.2)
	Selen whua HC	21 (15.7)	100 (74.6)	9 (6.7)	0 (0.0)		2 (1.5)	2 (1.5)

### Outcomes of anti-TB treatment

**Fig. 2:**
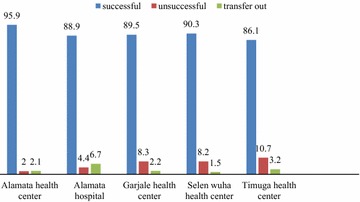
Treatment outcomes (expressed in percent) among health facilities. This shows variations in treatment outcomes across health facilities in the study district

The majority (90.1 %) had successful treatment outcomes, while 215 (5.0 %) had unsuccessful treatment outcomes [i.e. 35.3 % (76/215) defaulted, 58.6 % (126/215) died and 6.1 % (13/215) had treatment failure]. The overall rate of default was 1.8 %, with 61.8 % of the defaulting cases occurring among rural residents. Unsuccessful treatment outcomes were more frequent among patients who received re-treatment regimens than among new cases (18.2 versus 4.7 %, P < 0.001). Treatment outcomes also varied with disease classification; 10 % of patients with SPPTB had unsuccessful treatment outcomes as compared to 6.4 % of patients with SNPTB and 3.2 % of patients with EPTB (p < 0.001). Cure rate was 84.7 % among new cases and 69.8 % among re-treatment cases. The proportion of death was 5.1 % in SPPTB cases, 4.2 % in SNPTB cases, and 1.8 % in EPTB cases. A high proportion of the treatment failure cases was observed in the year 2011, which accounted for 53.8 % (Table 3). Treatment success varied from 86.1 to 95.9 % across the health facilities, with a 95.9 % treatment success obtained at the Alamata Health Center and 86.1 % at the Timuga Health Center (Fig. 2).

In the multivariable logistic regression model, there was significant heterogeneity in the odds of having unsuccessful treatment outcomes across years of initiating treatment and across health facilities in the study area (Table 4). Moreover, the odds of having unsuccessful treatment outcomes was significantly increased among SPPTB and SNPTB patients compared to EPTB cases, among HIV sero-positive patients compared to HIV sero-negative, and among retreatment cases compared to new cases (Table 4).

**Table 4:** Associations of selected background characteristics and the likelihood of having unsuccessful TB treatment outcomes (n = 4275)

Characteristics	Unsuccessful treatment outcomes		Crude odds ratio (95 % CI)	p value	Adjusted odds ratio (95 % CI)
	Yes	No			
Year of enrollment					
2007	28 (4.4)	614 (95.6)	0.5 (0.3, 0.9)	0.490	0.8 (0.3, 1.7)
2008	44 (5.2)	796 (94.8)	0.6 (0.4, 1.0)	0.730	0.9 (0.5, 1.7)
2009	36 (4.0)	856 (96.0)	0.5 (0.3, 0.8)	0.020	0.5 (0.3, .9)
2010	34 (4.3)	764 (95.7)	0.5 (0.3, 0.8)	0.010	0.5 (0.3, 0.8)
2011	47 (5.9)	743 (94.1)	0.7 (0.4, 1.2)	0.090	0.6 (0.4, 1.1)
2012	26 (8.3)	287 (91.7)	Reference		Reference
Health facilities					
Alamata hospital	109 (4.4)	2341 (95.6)	Reference		Reference
Alamata HC	18 (2.0)	883 (98.0)	0.4 (0.3, 0.7)	0.001	0.4 (0.3, 0.7)
Garjale HC	26 (8.3)	288 (91.7)	1.9 (1.2, 3.0)	<0.001	2.3 (1.5, 3.7)
Timuga HC	51 (10.7)	425 (89.3)	2.6 (1.8, 3.7)	<0.001	2.5 (1.8, 3.6)
Selen wuha HC	11 (8.2)	123 (91.8)	1.9 (1.0, 3.7)	0.090	1.8 (0.9, 3.5)
Site of illness					
SPPTB	59 (10.0)	530 (90.0)	3.4 (2.4, 4.8)	<0.001	3.1 (2.1, 4.5)
SNPTB	78 (6.2)	1171 (93.8)	2.0 (1.5, 2.8)	<0.001	2.1 (1.5, 2.9)
EPTB	78 (3.2)	2359 (96.8)	Reference		Reference
HIV					
Positive	52 (8.9)	535 (91.1)	2.1 (1.5, 3.0)	0.004	1.7 (1.2, 2.5)
Negative	101 (4.4)	2171 (95.6)	Reference		Reference
Unknown	62 (4.4)	1354 (95.6)	1.0 (0.7, 1.4)	0.660	0.9 (0.5, 1.6)
Treatment history					
New cases	195 (4.7)	3970 (95.3)	Reference		
Retreatment cases	20 (18,2)	90 (81.8)	4.5 (2.7, 7.5)	<0.001	2.6 (1.5, 3.7)
Sex					
Male	127 (5.0)	2395 (95.0)	1.0 (0.8, 1.3)	0.980	
Female	88 (5.0)	1665 (95.0)	Reference		
Age group					
0–14	24 (4.2)	541 (95.8)	0.8 (0.5, 1.3)	0.310	
15–24	39 (3.8)	985 (96.2)	0.7 (0.5, 1.1)	0.090	
25–34	55 (5.4)	958 (94.6)	1.0 (0.7, 1.5)	0.990	
35–44	44 (6.3)	653 (93.7)	1.2 (0.8, 1.8)	0.450	
45+	53 (5.4)	923 (94.6)	Reference		
Residence					
Urban	102 (5.0)	1951 (95.0)	1.0 (0.7, 1.3)	0.860	
Rural	113 (5.1)	2109 (94.9)	Reference		

*SPPTB* smear positive pulmonary TB, *SNPTB* smear negative pulmonary TB, *EPTB* extra pulmonary TB

## Discussion

Non-adherence to anti-TB treatment might lead to an increased risk of drug resistance and a prolonged infectiousness, in addition to relapse and death [19]. Thus, data obtained from this adherence survey is essential for planning, implementing and evaluating advocacy, communication and social mobilization (ACSM) work. Between November 2012 and January 2013, 200 TB patients who were on anti-TB drug treatment were interviewed using a structured questionnaire to evaluate level of adherence to anti-TB treatment in Alamata District, northeast Ethiopia. The results showed that an overall treatment adherence of 1 month was 88.5 %, which is higher than the level of adherence reported previously from Southwest Ethiopia [20]. The difference in adherence rates might be explained by the difference in the characteristics of study participants, as their study participants were only HIV positive patients, while the current study participants were a combination of those HIV seropositive and HIV seronegative. In contrast to the current study, a previous Ethiopian study [11] reported a significant difference in adherence to anti-TB treatment during the intensive and continuation phases of TB treatment. The present study demonstrated a similar level of non-adherence (11.5 %) to the results of studies from USA [21, 22] and China (12.2 %) [17], but lower than the results of previous studies in Ethiopia (26 %) [23], Zambia (22 %) [24], India (33 %) [25], Europe (31 %) [26] and Norway (17 %) [27]. On the other hand, higher level of non-adherence was observed in the current study compared to the non-adherence level (4.5 %) reported from Kenya [28].

In the current study, the main reasons for non-adherence were forgetting to take medication, being away from home, being unable to go to a health facility and negative drug side effects, which is consistent with the findings of previous Ethiopian studies [10–12]. In the present study, the proportion of non-adherence was high among frequent alcohol users and smokers, which supports the findings of a previous study [29]. These problems of non-adherence due to alcohol and smoking would be reduced by good communication between patients and health providers as well as by counseling. Previous evidence shows that good counseling [30] and good communication between the patient and health professionals [31] have a positive effect on patients’ adherence to their medication regimen.

Non-adherence to anti-TB treatment can adversely affect treatment success rate. Studies show that poor adherence to anti-TB treatment leads to treatment failure and death, while strict adherence helps to achieve the desired treatment success rate [32, 33]. In addition to the TB treatment adherence study, we assessed TB treatment outcomes in the present study area. The results showed that 90 % of patients had successful treatment outcomes, which is similar to the WHO target of 90 % (updated target 2011–2015) [34], though the treatment success rates varied from 86.1 to 95.9 % across the health facilities. The findings of the current study are comparable to the findings of another recent study in Ethiopia [14]. However, the proportion of successful treatment outcomes in the current study is relatively higher than findings reported from central Ethiopia [35], western Ethiopia [36], northwestern Ethiopia [37] and from other African countries including Tanzania [38], Nigeria [39] and Sudan [40].

The findings of the present study showed that among patients with unsuccessful treatment outcomes, the majority (58.6 %) had died. This may be associated with HIV, which has been recognized as the most important risk factor for unsuccessful TB treatment outcomes. The HIV epidemic has a multifaceted negative impact in the control of TB [41]. The findings of the present study also showed that HIV sero-positive patients were more likely to had unsuccessful treatment outcomes (52 out of 587, 8.9 %) compared to HIV sero- negative patients (101 out of 2272, 4.4 %).

This study showed that the overall default rate within the target study period was 1.8 %, which is much lower than the findings of previous studies in Ethiopia [9, 10, 42]. It is also lower than the WHO recommended default rate of 3 % [43]. This indicates that the present study area is on track for the achievement of the Millennium Development Goal focusing on the control of TB. It was also noted that patients on re-treatment (4.5 %) were more likely to default than new cases (1.7 %). This finding is in line with the findings of previous studies in Ethiopia [13, 14] and a study conducted in Morocco [3].

The treatment success of 83 % among SPPTB cases, in the current study is slightly low than the WHO targeted treatment success of 90 % for SPPTB cases. However, unsuccessful treatment outcomes were similar among urban and rural residents, which is inconsistent with the results of a previous retrospective study conducted in the Southern Region of Ethiopia, which reported that patients residing in urban areas had higher rates of unsuccessful treatment outcomes compared to patients from rural areas [13]. This difference may be due a difference in interventions and the duration of the study period.

### Strength and limitations of the study

In the anti-TB treatment adherence component of the current study, we attempted to ascertain the level of TB treatment adherence in all forms of TB patients who received their treatment at several health facilities in the study area and this could be considred as one of the strengths of the study. However, treatment adherence was assessed based on data from the past 1 month and patients’ self-report. Treatment adherence among younger patients (i.e. below 15 years of age) and those TB patients who followed their treatment at the health post level was not evaluated, which could be considered as the main limitations of the study. Similarly, treatment outcomes were assessed in all forms of TB patients who were treated at the different health facilities within 5 years and 6 months period. Nevertheless, it was not possible to determine all factors which could affect treatment outcomes since the results were based on secondary data which routinely collected from health facilities. Moreover, since the study did not include health posts it is difficult to generalize the findings of this study to all TB patients who are treated at health posts and other health facilities in the study area.

In conclusion, the level of adherence (88.5 %) observed in the current study area is relatively high. The treatment success rate (90 %) observed in the present study is also high. These are good practices in the study area, because effective treatment is not only an essential component of good patient care, but also a key element of the public health response to TB. However, still further effort like health education to patient or family is needed to reduce those factors which affect adherence and treatment success rates in order to ensure higher rates of adherence and treatment success in the present study area.

## References

[1] WHO (2012). Global tuberculosis report 2012. WHO/HTM/TB/2012,6.

[2] Volmink J, Garner P (2007). Directly observed therapy for treating tuberculosis. Cochrane Database of Syst Rev..

[3] Dooley KE, Lahlou O, Ghali I, Knudsen J, Elmessaoudi D, Cherkaoui I, Aouad RE (2011). Risk factors for tuberculosis treatment failure, default, or relapse and outcomes of retreatment in Morocco. BMC Publ Health.

[4] Charles P. Felton National Tuberculosis Center. Adherence to Treatment for Latent Tuberculosis Infection. A Manual for Health Care Providers. 2005.

[5] Franke F, Appleton C, Arteaga F, Palacios E, Llaro K, Shin S, Becerra C, Murray B, Mitnick D (2008). Risk factors and mortality associated with default from multidrug-resistant tuberculosis treatment. Clin Infect Dis.

[6] Kaona FA, Tuba M, Siziya S, Sikaona L (2004). An assessment of factors contributing to treatment adherence and knowledge of TB transmission among patients on TB treatment. BMC Publ Health.

[7] Ifebunandu NA, Ukwaja KN (2012). Tuberculosis treatment default in a large tertiary care hospital in urban Nigeria: prevalence, trend, timing and predictors. J Infect Publ Health.

[8] Muture BN, Keraka MN, Kimuu PK, Kabiru EW, Ombeka VO, Oguya F (2011). Factors associated with default from treatment among tuberculosis patients in Nairobi province, Kenya: a case control study. BMC Publ Health.

[9] Addis Z, Birhan W, Alemu A, Mulu A, Ayal G, Negash H (2013). Treatment outcome of tuberculosis patients in Azezo Health Center, North West Ethiopia. IJBAR.

[10] Gebremariam MK, Bjune GA, Frich JC (2010). Barriers and facilitators of adherence to TB treatment in patients on concomitant TB and HIV treatment: a qualitative study. BMC Publ Health.

[11] Shargie EB, Lindtjorn B (2007). Determinants of treatment adherence among smear positive pulmonary tuberculosis patients in Southern Ethiopia. PLoS Med.

[12] Tekle B, Mariam DH, Ali A (2002). Defaulting from DOTS and its determinants in three districts of Arsi Zone in Ethiopia. Int J Tuberc Lung Dis.

[13] Muñoz-Sellart M, Cuevas L, Tumato M, Merid Y, Yassin M (2010). Factors associated with poor tuberculosis treatment outcome in the Southern Region of Ethiopia. Int JTuberc Lung Dis.

[14] Berhe G, Enquselassie F, Aseffa A (2012). Treatment outcome of smear-positive pulmonary tuberculosis patients in Tigray Region, Northern Ethiopia. BMC Publ Health.

[15] Federal Ministry of Health, Ethiopia (FMoH) (2008). Manual for tuberculosis, leprosy and TB/HIV prevention and control program.

[16] CSA. Central stastical authority report of Ethiopia. Addis Ababa. 2008.

[17] Xu W, Lu W, Zhou Y, Zhu L, Shen H, Wang J (2009). Adherence to anti-tuberculosis treatment among pulmonary tuberculosis patients: a qualitative and quantitative study. BMC Health Serv Res..

[18] Federal Ministry of Health (FMoH). Guidelines for clinical and programmatic management of TB, leprosy and TB/HIV in Ethiopia. 5th edn. Addis Ababa, Ethiopia 2012.

[19] Haynes RB, Montague P, Oliveer T (2002). Interventions for helping patients to follow prescriptions for medications. Cochrane Database Syst Rev..

[20] Kebede A, Tajure WN (2012). Medication adherence and its determinants among patients on concomitant tuberculosis and antiretroviral therapy in south west Ethiopia. N Am J Med Sci.

[21] Bloch AB, Cauthen GM, Simone PM, Kelly GD, Dansbury KG, Castro KG (1999). Completion of tuberculosis therapy for patientsreported in the United States in 1993. Int J Tuberc Lung Dis.

[22] Jasmer RM, Seaman CB, Gonzalez LC, Kawamura LM, Osmond DH, Daley CL (2004). Tuberculosis treatment outcomes. Am J Respir CritCare Med.

[23] Nezenga ZS, Gacho YH, Tafere TE (2013). Patient satisfaction on tuberculosis treatment service and adherence to treatment in public health facilities of Sidama zone, South Ethiopia. BMC Health Serv Res.

[24] Mulenga C, Mwakazanga D, Vereecken K, Khondowe S, Kapata S, Shamputa C, Meulemans H, Rigouts L (2010). Management of pulmonary tuberculosis patients in an urban setting in Zambia: a patient’s perspective. BMC Publ Health.

[25] Gopi PG, Muniyandi MV, Chandrasekaran MV, Balasubramanian R, Narayanan PR (2007). Risk factors for non-adherence to directly observed treatment (DOT) in a rural tuberculosis unit, south India. India J Tuberc.

[26] Falzon D, Le Strat Y, Belghiti F, Infuso A (2005). Exploring the determinants of treatment success for tuberculosis cases in Europe. Int J Tubec Lung Dis.

[27] Farah MG, Tverdal A, Steen TW, Heldal E, Brantsaeter AB, Bjune G (2005). Treatment outcome of new culture positive pulmonary tuberculosis in Norway. BMC Publ Health.

[28] Nackers F, Huerga H, Espie´ E, Aloo A, Bastard M, Etard J, Sitienei J, Varaine F, Chakaya J, Bonnet M (2012). Adherence to self-administered tuberculosis treatment in a high HIV-prevalence setting: a cross-sectional survey in Homa Bay, Kenya. PLoS Med..

[29] Davidson H, Schluger NW, Feldman NW, Valentine DP, Telzak EE, Luefer FN (2000). The effects of increasing incentives on adherence to tuberculosis directly observed therapy. Int J Tuberc Lung Dis.

[30] Bello I, Itiol A (2010). Drug adherence amongst tuberculosis patients in the University of Ilorin Teaching Hospital, Ilorin, Nigeria. Afri J Phar Pharmacol.

[31] Simon L, Judy D, Merrick Z, Carl J (2005). Staff training and ambulatory treatment outcomes: a cluster randomized controlled trial in South Africa. Bull WHO.

[32] Burman WJ, Cohn DL, Rietmeijer CA, Judson FN, Sbarbaro JA, Reves RR (1997). Noncompliance with directly observed therapy for tuberculosis. Epidemiology and effect on the outcome of treatment. Chest.

[33] Ershova JV, Podewils LJ, Bronner LE, Slockwell HG, Dlamin S, Mametja LD (2014). Evaluation of adherence to national guidelines among tuberculosis patients in three provinces of South Africa. S Afr Med J..

[34] WHO. The global plan to stop TB, 2011–2015/Stop TB Partnership. Transforming the fight towards elimination of tuberculosis. Geneva. 2010.

[35] Getahun B, Ameni G, Medhin G, Biadgilign S (2013). Treatment outcome of tuberculosis patients under directly observed treatment in Addis Ababa, Ethiopia. Braz J Infect Dis.

[36] Demeke D, Legesse M, Bati J (2013). Trend of tuberculosis and treatment outcomes in Gambella Region with special emphasize on Gambella Regional Hospital, Western Ethiopia. J Mycobac Dis.

[37] Tessema B, Muche A, Bekele A, Reissig D, Emmrich F, Sack U (2009). Treatment outcome of tuberculosis patients at Gondar University Teaching Hospital, Northwest Ethiopia. A 5-year retrospective study. BMC Publ Health.

[38] van den Boogaard J, Lyimo R, Irongo CF, Boeree MJ, Schaalma H, Aarnoutse RE, Kibiki GS (2009). Community vs. facility-based directly observed treatment for tuberculosis in Tanzania’s Kilimanjaro Region. Int J Tuberc Lung Dis.

[39] Fatiregun AA, Ojo AS, Bamgboye AE (2009). Treatment outcomes among pulmonary tuberculosis patients at treatment centers in Ibadan, Nigeria. Ann Afr Med.

[40] El-Sony AI, Khamis AH, Enarson DA, Baraka O, Mustafa SA, Bjune G (2002). Treatment results of DOTS in 1797 Sudanese tuberculosis patients with or without HIV co-infection. Int J Tuberc Lung Dis.

[41] Godfrey-Faussett P, Ayles H (2003). Can we control tuberculosis in high HIV prevalence settings?. Tuberc.

[42] Shargie B, Lindtjørn B (2005). DOTS improves treatment outcomes and service coverage for tuberculosis in South Ethiopia: a retrospective trend analysis. BMC Publ Health.

[43] WHO. Framework for effective tuberculosis control. *WHO/TB/*1994, 179. Geneva. 1994.

